# Comparative performance of three experimental hut designs for measuring malaria vector responses to insecticides in Tanzania

**DOI:** 10.1186/s12936-016-1221-x

**Published:** 2016-03-15

**Authors:** Dennis J. Massue, William N. Kisinza, Bernard B. Malongo, Charles S. Mgaya, John Bradley, Jason D. Moore, Filemoni F. Tenu, Sarah J. Moore

**Affiliations:** Epidemiology and Public Health Department, Swiss Institute of Tropical and Public Health, Soccinstrase 57, 4002 Basel, Switzerland; University of Basel, Petersplatz 1, 4003 Basel, Switzerland; Amani Research Centre, National Institute for Medical Research, P. O. Box 81, Muheza, Tanga Tanzania; MRC Tropical Epidemiology Group, London School of Hygiene and Tropical Medicine, London, WC1E 7HT UK; Bagamoyo Research and Training Centre, Ifakara Health Institute, P.O. Box 74, Bagamoyo, Pwani Tanzania

**Keywords:** Experimental hut design, Malaria, Malaria vector, Insecticide, WHOPES, Long-lasting insecticidal net, Tanzania

## Abstract

**Background:**

Experimental huts are simplified, standardized representations of human habitations that provide model systems to evaluate insecticides used in indoor residual spray (IRS) and long-lasting insecticidal nets (LLINs) to kill disease vectors. Hut volume, construction materials and size of entry points impact mosquito entry and exposure to insecticides. The performance of three standard experimental hut designs was compared to evaluate insecticide used in LLINs.

**Methods:**

Field studies were conducted at the World Health Organization Pesticide Evaluation Scheme (WHOPES) testing site in Muheza, Tanzania. Three East African huts, three West African huts, and three Ifakara huts were compared using Olyset^®^ and Permanet 2.0^®^ versus untreated nets as a control. Outcomes measured were mortality, induced exophily (exit rate), blood feeding inhibition and deterrence (entry rate). Data were analysed using linear mixed effect regression and Bland–Altman comparison of paired differences.

**Results:**

A total of 613 mosquitoes were collected in 36 nights, of which 13.5 % were *Anopheles gambiae* sensu lato, 21 % *Anopheles funestus* sensu stricto, 38 % *Mansonia* species and 28 % *Culex* species. Ifakara huts caught three times more mosquitoes than the East African and West African huts, while the West African huts caught significantly fewer mosquitoes than the other hut types. Mosquito densities were low, very little mosquito exit was measured in any of the huts with no measurable exophily caused by the use of either Olyset or Permanet. When the huts were directly compared, the West African huts measured greater exophily than other huts. As unholed nets were used in the experiments and few mosquitoes were captured, it was not possible to measure difference in feeding success either between treatments or hut types. In each of the hut types there was increased mortality when Permanet or Olyset were present inside the huts compared to the control, however this did not vary between the hut types.

**Conclusions:**

Both East African and Ifakara huts performed in a similar way although Ifakara huts allowed more mosquitoes to enter, increasing data power. The work convincingly demonstrates that the East African huts and Ifakara huts collect substantially more mosquitoes than the West African huts.

## Background

The World Health Organization Pesticide Evaluation Scheme (WHOPES) was set up in 1960 to promote and coordinate the testing and evaluation of pesticides for public health. The scheme employs a four-phase testing programme to assess safety, efficacy and operational acceptability of public health pesticides to facilitate the registration of pesticides by the World Health Organization (WHO) member states [1]. Before they can receive approval from WHOPES, mosquito control interventions, including long-lasting insecticidal nets (LLINs) [1] and indoor residual spray (IRS), are evaluated for their vector control efficacy in Phase II experimental hut studies. Four indicators are normally used to assess the efficacy of formulated insecticides used in IRS or in LLINs: (1) deterrence (entry rate): refers to the total number of female mosquitoes found in the hut and exit traps relative to the control due to being deterred from entry into treated huts by the presence insecticide; (2) induced exophily (exit rate): the proportion of female mosquitoes found in the exit traps compared with the total number found in the hut (including traps) where mosquitoes may be irritated by the presence of insecticide and are therefore more likely to leave; (3) the reduction in blood feeding in comparison with control, although for the purposes of this comparison we used feeding success, i.e., the proportion of blood-fed mosquitoes captured in the hut; and, (4) mortality: proportion of dead female mosquitoes found in the hut after collection and holding for 24 h. If the performance of a new product is equivalent to or exceeds the efficacy of a gold standard product with WHOPES recommendation in field tests, and also passes critical thresholds of 80 % mortality and 95 % knockdown in cone bioassays over a period of time (for IRS) or number of washes (for LLINs), then the product receives interim recommendation from WHOPES and can be included in large-scale phase III field trials under user conditions, with a larger sample size required for decision making on full recommendation for use in vector control programmes.

There are currently three kinds of experimental huts included in WHOPES guidelines for testing of insecticidal products [2]: West African huts, East African huts and Asian style huts. In addition, there are currently three other styles of portable huts being used for evaluation of insecticides including behavioural measures of efficacy in South America [3], Thailand [4] and Tanzania [5]. Although these huts have been in use for many years [6], their comparative performance in measuring the key entomological parameters required for decision making by WHOPES has not been evaluated. In this paper an experimental comparison of the three kinds of huts in use in Africa was conducted in order to compare their performance in assessing the efficacy of insecticide used in LLINs in Tanzania.

## Methods

### Study area and experimental huts

Field studies were conducted in nine experimental huts at Zeneti village in Muheza District, northeast Tanzania (5° 13′S, 38° 39′E, altitude 193 m) where the National Institute of Medical Research (NIMR) Muheza experimental huts are located. *Anopheles gambiae* sensu stricto, *Anopheles arabiensis*, *Anopheles funestus s.s.* and *Culex quinquefasciatus* are the predominant mosquito species in the area (Nkya, unpublished data). The experimental huts consisted of three East African hut design (constructed according to a design first described by Smith [7]), three West Africa hut design [8] and three Ifakara hut design [5] (Table 1), and were situated near rice and vegetable fields, arranged in two rows with a 5-m gap between the huts. For six nights before the start of the experiments, collections were conducted with sleepers, but no interventions, in the huts to ensure that there was no significant bias between attractiveness of huts or sleepers to mosquitoes.

**Table 1:**
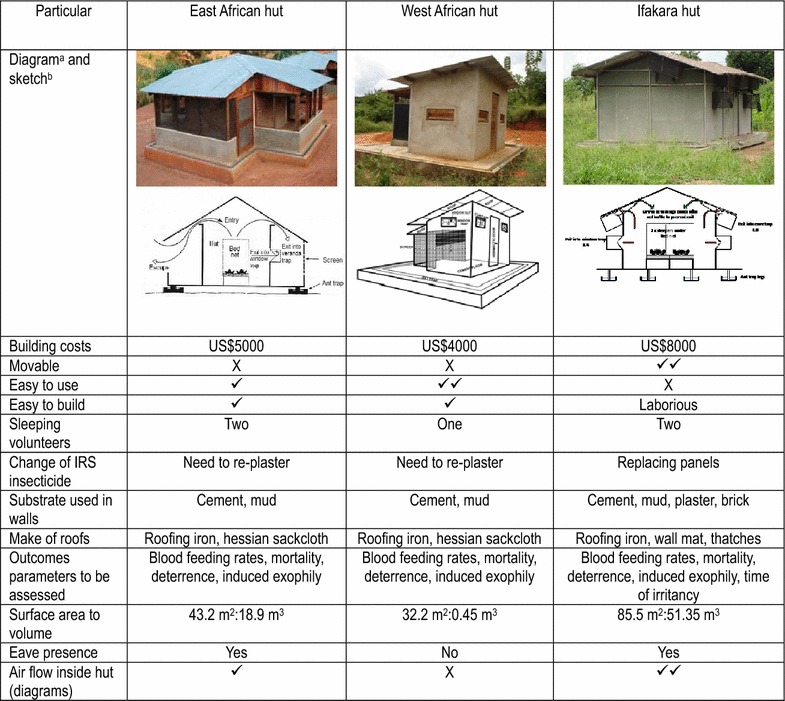
Main design characteristics of the East African, West African and Ifakara experimental huts

^a^Ifakara hut hut-courtesy of Dr. Sarah Moore, Ifakara Health Institute, Tanzania

^b^East African hut design-courtesy of Late Professor C.F. Curtis; West African Hut-courtesy of Dr. J.M. Hougard, Benin; Ifakara hut-courtesy of Dr. Sarah Moore, Tanzania

### Treatment, sleepers and mosquito collections

Each of the three hut types was evaluated by a separate 3 × 3, balanced, partially randomized, Latin square design that was repeated four times so that each hut type was assigned each treatment 12 times over 36 collection nights conducted over 6 weeks between 20 July and 1 September 2011, during the dry season. Each of the nine huts was assigned one of three bed net treatments: (1) Olyset^®^ 2.0 (A to Z Textile Mills, Arusha, Tanzania); (2) Permanet^®^ 2.0 (Vestergaard-Fransden); and, (3) untreated Safi Net (A to Z Textile Mills, Arusha, Tanzania). Treatments were rotated after every three nights of experiments.

At the end of each three-night experiment round the huts were cleaned and aired for 1 day to prevent carry-over insecticide residuals and the treatment moved to the next hut. Fifteen adult men (two for each of the East African hut design and Ifakara hut design and one for each of the West African hut design) volunteered to sleep in the huts from 18.00 to 06.30 h and to collect mosquitoes in the mornings. The sleepers were experienced in collecting mosquitoes and were assigned to one hut type for the duration of the experiment, then rotated between the three huts of that type on a nightly basis to remove bias associated with differential attractiveness of humans to mosquitoes. Each morning at 06.30 the huts were searched and all mosquitoes were collected from the floors, walls and ceilings of rooms, verandah/exit traps and inside of mosquito nets using a mouth aspirator and torch, and placed in paper cups labelled by date, hut, treatment, and trap types. Data collection followed standard operating procedures (SOP) developed for the experiment based on WHO guidelines [2]. Mosquitoes were sorted, counted, identified morphologically to genus level, scored as dead or alive and *An. gambiae s.l.* were scored as blood fed or unfed. Live mosquitoes were held for 24 h in netted plastic cups supplied with 10 % glucose solution to monitor delayed mortality. Male mosquitoes were not scored. After 24 h any live mosquitoes were killed and female *Anopheles* identified to species level [9, 10]. Molecular analysis by polymerase chain reaction (PCR) [11] was used to distinguish between specimens of the *An. gambiae* species complex. PCR was not conducted for *An. funestus* as only *An. funestus s.s.* is present in the study area.

### Outcome measurements

The impact of each treatment was assessed according to the following parameters: (1) deterrence (entry rate): percentage reduction in the number of mosquitoes caught in treated hut relative to the number caught in the control hut; (2) induced exophily (exit rate): the percentage of female mosquitoes found in the exit traps compared with the total number found in the hut and traps. The reduction of the exit rate allows estimation of induced exophily or exito-repellency; (3) blood-feeding success: proportion of fed female mosquitoes compared with the total number found in the hut. The reduction in the number of blood-fed mosquitoes between a treated hut and a control hut allows an assessment of the blood-feeding inhibition caused by insecticide; and, (4) mortality: percentage of dead female mosquitoes found in the hut immediately after and 24 h later. The difference in mortality between a control hut and a treated hut allows assessment of the insecticide-induced mortality. Mortality was also corrected for control using Abbotts formula [12].

### Data management and analysis

Data were collected in standardized field-data forms and entered into Microsoft Excel. JB and SJM conducted the analysis blinded to the treatment allocation through coding by DJM. Data were cleaned in STATA 11 (StataCorp, College Station TX, USA) by checking for balance, outliers and unusual observations through tabulation and graphing. Data were analysed using STATA and the R Statistical software version 2.15.0 [13] with significance level of 0.05 for rejecting the null hypothesis following a predefined analysis plan. All mixed models in R were conducted using the lme4 package [14]. Count data (deterrence) were modelled using a generalized linear mixed model (GLMM) with a log link and a Poisson distribution with position, sleeper, and day of experiment fitted as random effects, and an intercept for each observation to model over-dispersion; treatment and hut type were fixed effects and these two factors were also modelled with an interaction, although this did not give the best model fit and was not used as the final model. Proportional data (mortality, induced exophily, blood-feeding inhibition) were analysed using GLMM with a logit link and a binomial distribution with the factors, hut location and day of experiment, fitted as random effects and an intercept for each observation to model over-dispersion; treatment, hut type were fixed effects with treatment and hut type. The interaction between hut and net type were fitted in one of the models although the final model did not have an interaction between these two factors. Several GLMMs were run for each outcome and the final model selected was that with the lowest Akaike’s information criterion (AIC). In addition, residuals were plotted using histogram, quintile plots and comparison with fitted values to ensure appropriateness of model selection. Ninety-five per cent confidence interval (CI) of adjusted odds ratio (OR) or incidence rate ratio (IRR) were also calculated using appropriate regression model for the differences between huts.

In order to see if there was a systematic difference between hut types, the extent of agreement in the total numbers of mosquitoes and of *An. gambiae s.l.* caught by each hut type when the same treatment was used was assessed by Bland–Altman methods [15] through the Batplot function in Stata 11. On each night, each of the three net types was tested in each of the three hut types. The count of mosquitoes captured each night in each hut type/net combination was compared pair-wise (although volunteers could not be fixed for a direct comparison which does introduce a bias). Data were log transformed and compared to see if the paired differences were dependent on mosquito density [16]. In addition, Bland–Altman agreement was measured using a one-sample t test to compare the differences of the two measurements to zero, and a linear regression of the paired differences against the average of the two methods, again in Stata.

### Ethical issues

Volunteers were recruited on a voluntary basis and signed a written informed consent form. The risks and benefits of the study were clearly explained, and volunteers were free to leave at any time during the study. Volunteers were provided with clothing to protect them from the cold temperature at night and were advised to dress in shorts that reached the knees with covered shoes to avoid bites on the feet. They were required not to smoke, take alcohol or use scented soaps and deodorants 6 h prior to experiments. Adverse events such as respiratory symptoms were monitored. The participants were also compensated for their time. The ethical review boards of Ifakara Health Institute IHI/IRB/No A-019-2007, the Medical Research Coordinating Committee of the National Medical Research Institute Tanzania (NIMR/HQ/R.8c/Vol. 1/160) approved the study.

## Results

A total of 613 mosquitoes were recorded from all huts over 36 nights, of which 13.5 % were *An. gambiaes.l.,* 21 % *An. funestus*, 38 % *Mansonia* species, and 28 % *Culex* species. The low density of *An. gambiaes.l.* was due to the huts only being available for use in the dry season. PCR analysis showed that 65 % (45/69 successful amplifications) of *An. gambiae* were *An. gambiae s.s.* and the remaining 35 % (24/69 successful amplifications) were *Anopheles arabiensis.* The relative proportion of *An. gambiae* sub-species collected from the East African huts and the Ifakara huts was consistent: East African huts collected 67 % *An. gambiae s.s.* and 33 % *An. arabiensis* while the Ifakara huts collected 66 % *An. gambiae s.s.* and 34 % *An. arabiensis.* There was no *An. gambiaes.s.* caught and only one *An. arabiensis* mosquito was collected from the West African huts. There were consistent trends in the way in which huts met the standard WHO criteria used to evaluate LLINs.

### Deterrence

**Fig. 1:**
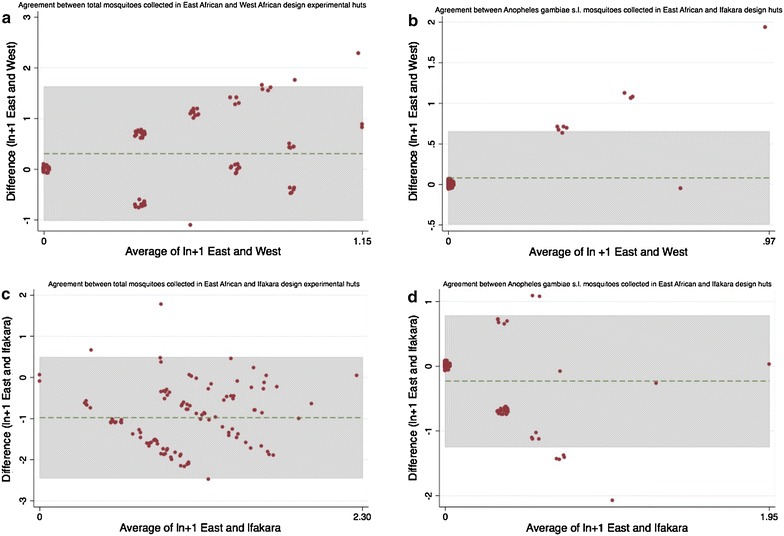
Bland–Altman comparison of difference in measurements made by East African experimental huts compared with West African huts and Ifakara huts using data transformed by natural log +1. **a** Total mosquitoes captured in East African and West African huts; **b** *Anopheles gambiae s.l.* captured in East African and West African huts; **c** Total mosquitoes captured in East African and Ifakara huts; **d** *Anopheles gambiae s.l.* captured in East African and Ifakara huts

In each of the three hut types there was no significant deterrence measured by the use of either Olyset or Permanet 2.0 LLINs (Table 2). When the number of mosquitoes caught in each of the three hut types was analysed by intervention there were clear trends (Table 3). The Ifakara hut caught between three and four times more mosquitoes than the East African hut, regardless whether the hut contained an untreated net or either type of LLIN, which was highly statistically significant in each case (p < 0.0001). The West African huts caught significantly fewer mosquitoes than the East African huts regardless of the intervention in the hut, ranging from IRR of 0.21 with control to 0.48 with Olyset, and again the IRR was highly significant. Exploration of the data using Bland–Altman methods highlighted a systematic density-dependent difference between the numbers of mosquitoes that the huts caught (Fig. 1 and Table 4). As mosquito densities increased, the East African huts consistently caught more mosquitoes than the West African huts and the Ifakara huts consistently caught more than the East African huts. The differences were all greater than zero by a one-sample t test, suggesting that the huts were different in their sampling efficiency (Table 4). For this reason the data were transformed using natural log +1 to account for zeros in line with the recommendations of Bland and Altman [15]. This transformation removed much of the density-dependent difference between the measurements (Fig. 1 and Table 5) although a one-sample t test was significantly different from zero, suggesting that the huts remained different in their sampling efficiency even though much of the variability in the data was removed.

**Table 2:** Deterrence caused by Olyset, Permanet 2.0 long-lasting insecticidal nets plus untreated control in East African, Ifakara and West African experimental huts—total mosquitoes

	N *An. gambiae s.l.*	N *An. funestus s.l.*	N All mosquitoes	Median IQR All mosquitoes	Incidence rate ratio (IRR)	95 % CI	p	z	% Deterrence *An. gambiae s.l.*	% Deterrence all mosquitoes
East African										
Control	9	18	42	1 (0–1.5)	1				–	–
Permanet 2.0	6	19	39	1 (0–2)	0.95	0.08–10.94	0.824	−0.222	33	7
Olyset^®^	3	18	33	1 (0–1)	0.78	0.11–8.67	0.297	−1.043	66	21
Ifakara										
Control	33	20	149	4 (2–6)	1				–	–
Permanet 2.0	10	16	140	3 (2–6)	0.88	0.10–7.94	0.269	−1.104	70	6
Olyset^®^	20	29	161	4 (3–5.5)	1.01	0.11–9.05	0.910	0.113	40	0
West African										
Control	0	0	8	0 (0–0)	1				–	–
Permanet 2.0	0	3	16	0 (0–1)	1.78	0.09–34.49	0.165	1.388	NA	11
Olyset^®^	1	3	16	0 (0–1)	1.78	0.09–34.50	0.165	1.389	NA	11

The IRR is calculated from Poisson regression as the incidence of mosquitoes in the treatment relative to the control

**Table 3:** Measurements of deterrence compared between East African, Ifakara and West African experimental huts for three treatment arms: Olyset, Permanet 2.0 long-lasting insecticidal nets plus untreated control

Count	All mosquitoes N	All mosquitoes median IQR	Incidence rate ratio (IRR)	95 % CI	p	z
Control						
East African	42	1 (0–1.5)	1			
Ifakara	149	4 (2–6)	3.79	0.37–38.87	<0.0001	7.719
West African	8	0 (0–0)	0.21	0.01–3.61	<0.0001	−4.219
Permanet						
East African	39	1 (0–2)	1			
Ifakara	140	3 (2–6)	3.50	0.34–36.42	<0.0001	7.029
West African	16	0 (0–1)	0.39	0.03–5.55	0.0018	−3.116
Olyset						
East African	33	1 (0–1)	1			
Ifakara	161	4 (3–5.5)	4.88	0.46–52.18	<0.0001	8.344
West African	16	0 (0–1)	0.48	0.03–6.88	0.016	−2.391

The IRR is calculated from Poisson regression as the incidence of mosquitoes in the treatment relative to the control

**Table 4:** Bland–Altman comparisons of total mosquitoes and total *Anopheles* *gambiae* *s.l.* caught in West African and Ifakara experimental huts compared to East African experimental huts

East African compared to	West African		Ifakara	
	All mosquitoes	*An. gambiae s.l.*	All mosquitoes	*An. gambiae s.l.*
Bland–Altman statistics				
Mean difference	0.685	0.157	−3.204	−0.417
Limits of agreement	−2.366, 3.736	−1.187, 1.502	−8.804, −2.396	−0.621, −0.213
T test statistics				
T test df	107	107	107	107
T test mean	0.685	0.157	−3.204	−0.4167
T test 95 % CI	0.388, 0.982	0.266, 0.288	−3.749, −2.659	−0.621, −0.213
T statistic	4.5742	2.3853	−11.6525	−4.0511
P value mean = 0	<0.0001	0.0188	<0.0001	0.0001
Regression statistics				
Regression df	107	107	107	107
R squared	0.49	0.92	0.31	0.24
Coefficient	1.46	1.86	−1.02	−0.68
t	10.14	36.47	−6.95	−5.72
p	<0.0001	<0.0001	<0.0001	<0.0001

**Table 5:** Bland–Altman comparisons of natural log +1 total mosquitoes and natural log +1 total *Anopheles gambiae* *s.l.* caught in West African and Ifakara experimental huts compared to East African experimental huts

East African compared to	West African		Ifakara	
	All mosquitoes	*An. gambiae s.l.*	All mosquitoes	*An. gambiae s.l.*
Bland–Altman statistics				
Mean difference	0.309	0.081	−0.978	−0.228
Limits of agreement	−1.001, 1.625	−0.491, 0.652	−2.444, 0.488	−1.242, 0.785
T test statistics				
T test df	107	107	107	107
T test mean	0.309	0.081	−0.978	−0.2284
T test 95 % CI	0.181–0.437	0.249, 0.136	−1.121, −0.836	−0.3270, −0.1297
T statistic	4.7783	2.8719	−13.5932	−4.5911
P value mean = 0	<0.0001	0.0049	<0.0001	0.0001
Regression statistics				
Regression df	107	107	107	107
R squared	0.15	0.82	0.75	0.21
Coefficient	0.80	1.67	0.06	−0.76
t	4.48	22.25	0.32	−5.23
p	<0.0001	<0.0001	0.747	<0.0001

After transformation, East African huts captured 0.8 more mosquitoes than West African Huts for every average increase in one mosquito of any species (R^2^ = 0.15, p < 0.0001), and 1.67 more *An. gambiae* for every increase in density of one *An. gambiae* mosquito (R^2^ = 0.82, p < 0.0001). This suggests a greater efficiency of the East African huts versus West African huts for capturing *An. gambiae s.l.* in the Tanzanian setting.

After transformation, East African and Ifakara huts had similar efficiency in the total number of mosquitoes collected, with a non-significant difference seen by regression of the differences *versus* the average of mosquitoes collected by the two hut types (R^2^ = 0.75, p = 0.747). The Ifakara huts were consistently more efficient in collecting *An. gambiae s.l.* with the East African huts collecting 0.76 fewer *An. gambiae* than the Ifakara huts for every increase in one *An. gambiae* in the environment (R^2^ = 0.21, p < 0.0001). However, it is cautioned that if the R^2^ statistic is quite small, then further data collection when mosquito numbers are higher is warranted to make a more accurate estimation of the relative efficiency of the two hut types.

The reason for this difference in mosquito densities between huts is most likely due to hut design and operation. Each of the West African had one volunteer inside and therefore has 50 % fewer host cues (carbon dioxide, fatty acids, heat, water vapour) emanating from it to attract mosquitoes, and had an area available for mosquito entry that was 80 % smaller than the East African huts. The Ifakara huts have 13 times greater area for mosquito entry than East African huts (Table 1).

### Excito-repellency (induced exiting)

As mosquito densities were so low, very little mosquito exit was measured in any of the huts (Tables 6 and 7) and no increase in exit caused by the use of either Olyset or Permanet was measurable. However, when the huts were compared side by side (Table 7) for control, Permanet or Olyset, there was far greater exophily measured by the West African huts. This is likely due to the fact that West African huts have a large exit gap available to mosquitoes (Table 1). The ratio of exit area available in West African huts is 50 times greater than in East African huts and 13 times greater than in Ifakara Huts.

**Table 6:** Induced exophily caused by insecticidal interventions: Olyset, Permanet 2.0 long-lasting insecticidal bed nets plus untreated control in East African, Ifakara and West African experimental huts

	Geometric mean exiting (all mosquitoes)	OR	95 % CI	p	z	Exophily % (95 % CI)^a^
East African						
Control	1 (1–1)	1				7.11 (2.26–11.99)
Permanet 2.0	0 (^a^)	0.32	0.0005–207.03	0.34	−0.948	2.50 (−1.35–6.34)
Olyset^®^	1 (^a^)	0.39	0.0006–243.30	0.42	−0.798	2.95 (−1.41–7.31)
Ifakara						
Control	1.49 (1.05–2.09)	1				6.91 (0.8–13.73)
Permanet 2.0	1.59 (1.09–2.31)	1.07	0.04–24.64	0.89	0.138	7.12 (0.36–13.88)
Olyset^®^	1.12 (0.83–1.51)	0.62	0.02–16.34	0.35	−0.935	4.34 (−1.47–10.15)
West African						
Control	1.26 (0.47–3.40)	1				50.00 (31.29–68.71)
Permanet 2.0	1 (1–1)	0.21	0.001–42.82	0.12	−1.560	18.69 (8.43–28.95)
Olyset^®^	1 (1–1)	0.27	0.001–51.29	0.19	−1.317	25.00 (12.43–13.73)

^a^95 % CI calculated using 40 df

**Table 7:** Measurements of induced exophily compared between East African, Ifakara and West African experimental huts for three treatment arms: Olyset, Permanet 2.0 long-lasting insecticidal bed nets plus untreated control

Exophily	OR	95 % CI	p	z	Exophily % (95 % CI)^a^
Control					
East African	1				7.11 (2.26–11.99)
Ifakara	0.92	0.02–46.05	0.91	−0.118	6.91 (0.8–13.73)
West African	14.45	0.0008–2582.05	0.006	2.745	50.00 (31.29–68.71)
Permanet 2.0					
East African	1				2.50 (−1.35–6.34)
Ifakara	3.03	0.009–934.13	0.30	1.033	7.12 (0.36–13.88)
West African	9.45	0.001–7074.31	0.06	1.846	18.69 (8.43–28.95)
Olyset					
East African	1				2.95 (−1.41–7.31)
Ifakara	0.85	0.01–6306.70	0.72	0.360	4.34 (−1.47–10.15)
West African	10.39	0.004–548.50	0.05	1.976	25.00 (12.43–13.73)

^a^95 % CI calculated using 40 df

### Feeding success

As unholed nets were used in the experiments and so few mosquitoes were captured, it was not possible to measure difference in feeding success either between treatments or hut types.

### Induced mortality

In each of the hut types there was increased mortality when Permanet or Olyset were present inside the huts compared to the control, however this was not statistically significant in any of the hut types, possibly due to the extremely high control mortality in each of the hut types (Tables 8 and 9). It is normal for data to be discarded if control mortality exceeds 20 %, so it is not possible to make any inference on the efficacy of the LLINs tested or any differences between measurements made in different hut types.

**Table 8:** Mortality caused by insecticidal interventions: Permanet 2.0, Olyset long-lasting insecticidal bed nets plus untreated control in Ifakara and West African experimental huts

	Dead (all mosquitoes)	Median IQR dead	OR	95 % CI	p	z	Mortality % (95 % CI)	Control corrected mortality %
East African								
Control	16	0 (0–1)	1				41.39 (20.17–62.61)	
Permanet 2.0	19	0 (0–1)	1.43	0.04–49.86	0.551	0.597	48.73 (27.05–70.40)	12.52
Olyset^®^	16	0 (0–1)	1.37	0.04–51.04	0.611	0.508	42.50 (21.67–63.32)	1.89
Ifakara								
Control hut	58	1 (0–3)	1				38.08 (25.64–50.52)	
Permanet 2.0	67	1 (0–3)	1.53	0.11–20.83	0.1329	1.503	44.93 (32.68–57.19)	11.06
Olyset^®^	72	2 (1–3)	1.47	0.11–19.84	0.1665	1.384	46.14 (37.04–55.25)	13.02
West African								
Control	5	0 (0–0)	1				50 (3.76–96.24)	
Permanet 2.0	11	0 (0–0.5)	2.88	0.01–841.14	0.321	0.993	100 (100–100)	100
Olyset^®^	10	0 (0–0.5)	2.00	0.01–534.15	0.508	0.662	100 (100–100)	100

**Table 9:** Measurements of mortality compared between East African, Ifakara and West African experimental huts for three treatment arms: Olyset, Permanet 2.0 long-lasting insecticidal bed nets plus untreated control

Mortality	Dead (all mosquitoes)	OR	95 % CI	p	z	Mortality % (95 % CI)	Control corrected mortality %
Control							
East African	16	1	–	–	–	41.39 (20.17–62.61)	
Ifakara	58	0.87	0.04–21.45	0.784	0.274	38.08 (25.64–50.52)	
West African	5	1.63	0.01–247.02	0.599	0.525	50 (3.76–96.24)	
Permanet							
East African	19	1	–	–	–	48.73 (27.05–70.40)	12.52
Ifakara	67	0.90	0.03–21.73	0.828	0.218	44.93 (32.68–57.19)	11.06
West African	11	3.29	0.05–237.93	0.127	1.526	100 (100–100)	100
Olyset							
East African	16	1	–	–	–	42.50 (21.67–63.32)	1.89
Ifakara hut	72	0.94	0.04–23.91	−0.114	0.909	46.14 (37.04–55.25)	13.02
West African	10	2.41	0.04–165.71	1.142	0.253	100 (100–100)	100

## Discussion

This study directly compares the performance of the three experimental huts in measuring the key entomological parameters. Although the total numbers of *An. gambiaes.l.* collected from the East African huts and Ifakara huts were low and the hut design and position of each hut type were different, the proportion of mosquitoes of each species caught was consistent between the East African and Ifakara huts. This consistent trend means that either of the East African and Ifakara experimental hut types can be used interchangeably to sample malaria vectors and measure standard WHO criteria used in evaluation of LLINs, and data are more comparable if transformed using a natural log +1. However, the Ifakara hut caught around four times more mosquitoes than the East African hut as it has a large area available for mosquito entry, which is a useful feature when mosquito density is important for increasing the power or precision in evaluation of mosquito control tools [17]. West African huts caught extremely low numbers of mosquitoes, making them unsuitable for evaluation of insecticidal tools in this setting. All three hut types showed a density-dependent effect, with the East African huts collecting consistently more mosquitoes than the West African huts, and the Ifakara huts collecting more than the East African huts as mosquito densities increased. It is likely that this is related to the surface area of the huts available for mosquito entry, with West African huts having 0.2 of the surface area for entry and Ifakara huts 13 times the surface area for entry relative to the East African huts.

The East African huts at this study site do not have baffles [18] and therefore mosquitoes could enter and leave the hut at any side. However, during the night of experiment, the verandah traps on two opposing sides were left open while the other two were closed to capture any mosquito that tried to exit. The number of mosquitoes collected each night in the two verandah traps was multiplied by two and added to the room and window/exit trap collections. This multiplication was done to adjust for the unrecorded escapes through the two verandahs, which were left unscreened to allow routes for entry of wild mosquitoes via the gaps under the eaves [19].

Data from Ifakara huts on the efficacy of eave baffles in preventing eave egress showed that the presence of baffles increased the likelihood of *An. arabiensis* being trapped in a window exit trap by around 50 % (RR (95 % CI) = 1.57 (1.03–2.37), z = 2.13, p = 0.033) and tripled the likelihood of *An. arabiensis* being trapped in an eave exit trap (RR = 2.90, p = 0.0001). Baffles increased the overall number of *An. arabiensis* collected by about 50 % (RR 1.44 p = 0.001) [5]. The deterrent property of insecticidal tools such as LLINs and IRS measured by different hut designs varied due to the possibility of mosquitoes escaping or being lost (e.g., through predation) and the total number of mosquitoes caught in the experimental hut generally underestimates the number of mosquitoes that entered. The number escaping will be higher with increased excito-repellence, but lower with increased insecticidal effect [20].

The West African huts measured greater excito-repellency than the East African and Ifakara huts due to the large exit gap (verandah) present for mosquitoes. Even the control had 50 % egress of mosquitoes. Similar results were also observed by Koudou and colleagues in Côte d’Ivoire [21] in which significant number of mosquitoes were caught in exit traps (verandah) of huts with treated nets compared to huts with control nets. Intact nets (absence of holes in nets) and the irritant effect of pyrethroids could have impacted not only on the low mosquito density but also low blood feeding success in any of the huts or treatments. This was due to the fact that the treated nets prevented the access of mosquitoes to blood meals. Surprisingly, exit from the Ifakara huts was lower than measured in another study using unholed Permanet 2.0 and Olyset nets [22] but data were similar in both the East African and the Ifakara huts for induced exophily from each of the interventions. This lower exophily may be because the control mortality was unacceptably high at 40 % in each of the hut types, most likely related to the harsh weather conditions and possibly exacerbated by poor handling. It is accepted that exophily is less likely to be recorded when mortality is higher, simply because the dead mosquitoes in the huts cannot escape [20].

All the three experimental huts recorded relatively high mortality when treated nets (Olyset or Permanet 2.0) were used. However, this was not statistically significant and could not be adequately interpreted because of the high control mortality in the study reported here and is contrary to findings from previous studies conducted in Muheza, Tanzania [19], Ifakara, Tanzania [22], and in Yaokoffikro, Côte d’Ivoire [21] where the mortality in huts with treated nets was significantly higher than in huts with untreated nets, and control was below the agreed acceptable standard of 10 %. This underlines the importance of careful monitoring of control mortality on a daily basis and repetition of experiments should control mortality exceed 10 %.

This study has a number of weaknesses. Due to other projects running at the site, a window to conduct the study became available only when mosquito numbers were low and climatic conditions may have raised mosquito mortality. A second weakness was the fact that the 24-h mortality was calculated for total mosquitoes and not for *Anopheles* mosquitoes. This study should have been repeated and closely monitored to ensure control mortality was at an acceptable level (<10 %). Therefore, it was not possible to measure the effect of the interventions on the target malaria vector species and compare these between hut types. It highlights the challenges of working in experimental huts where studies may need to be performed for long periods of time in order to collect sufficient numbers of mosquitoes to discriminate between treatments, and the careful oversight needed for studies to be conducted to a high standard. While these are limitations, it can clearly be seen through the side-by-side evaluation used that the East African huts and Ifakara huts had greater comparability than East African and West African huts in this setting.

## Conclusions

Both East African huts and Ifakara huts performed in a similar way although Ifakara huts allowed more mosquitoes to enter, increasing data power. The work convincingly demonstrates that the Ifakara and East African huts collect substantially more mosquitoes than West African huts. Unfortunately, mortality and blood feeding rates, probably the two most crucial outcomes, could not be assessed due to high mortality and low numbers of blood-fed mosquitoes in the controls; hence, the controversy of how well the huts perform in terms of product evaluation remains unresolved, although it is conceivable that huts attracting more mosquitoes would yield more sensitive measurements.
